# A co-produced method to involve service users in research: the SUCCESS model

**DOI:** 10.1186/s12874-019-0671-6

**Published:** 2019-02-15

**Authors:** Bridie Angela Evans, Alison Porter, Helen Snooks, Vanessa Burholt

**Affiliations:** 1 Medical School, Swansea University, Singleton Park, Swansea, SA2 8PP UK; 2 Centre for Innovative Ageing, College of Human and Health Science, Swansea University, Swansea, UK

**Keywords:** PPI, Patient and public involvement, Co-production

## Abstract

**Background:**

Public and patient involvement is a routine element of health services research methods to produce better designed and reported studies. Although co-production is recommended when involving people in research, methods for involving people are usually designed and managed by researchers and there is little evidence about methods to co-produce models for effective public and patient involvement. We report the method used by a group of patient and carer service users to develop and implement a model for involving public members in research.

**Method:**

We recruited people with experience of chronic conditions, as patients and carers, and supported them to develop and implement the involvement model. We collected written records to describe the processes of co-production.

**Results:**

Sixteen service users were involved through a series of workshop, meeting and email discussions. They specified principles and operating characteristics of the model which concerned an inclusive culture, adequate resources, accessibility, good communication and clarity of purpose and roles. Components of the model included an on-line Panel of members (*n* = 20), Steering Group meetings, representation and communication system, facilitator, supportive research environment and access to research activities. Over 8 years, members were active in 218 research activities and held 22 Steering Group meetings. The model was named SUCCESS standing for Service Users with Chronic Conditions Encouraging Sensible Solutions.

**Conclusion:**

We supported patients and carers to co-produce the SUCCESS model of involvement in research. The model’s components, addressing their needs and priorities, led to sustained involvement in research over 8 years. Further work is needed to apply the model in different settings and assess impact of this method of involving people in research.

**Electronic supplementary material:**

The online version of this article (10.1186/s12874-019-0671-6) contains supplementary material, which is available to authorized users.

## Background

Public and patient involvement is a routine element of health services research in order to make research more accountable, rigorous and relevant [1–4]. There is evidence that research methods planned and implemented in partnership with patients and public members produce better designed and reported studies [5, 6]. Involving patients and public members in designing and carrying out studies and disseminating findings is seen as a way to narrow the gap between patient and clinician priorities, to change the focus of intervention development and implementation [7–10] and to improve implementation of evidence-based findings [11]. The National Institute for Health Research (NIHR) expects researchers to demonstrate public involvement in proposals seeking NIHR funding in order to enhance research quality [12]. The UK goal is for all patients and more public members to be aware of and involved in research by 2025 [13]. Researchers are increasingly following this method of designing and undertaking research and the number of studies which involve public members is steadily rising [14, 15].

Meaningful public involvement in research is best achieved by collaboration, where research is co-produced by public and patient members working within research teams, or by service user-led approaches [1, 16, 17]. The aim is to enable insight, derived from people’s experiences of a health condition or care service, to shape the questions which are defined and how they are studied by research teams [5, 18, 19]. This fits the definition of active involvement, as research carried out ‘with’ or ‘by’ members of the public, rather than ‘to’, ‘about’ or ‘for’ them [1].

Methods for involving patients and public members in research are usually designed and managed by researchers who have access to guidance and others’ experience about how to achieve collaborative working [1, 20, 21]. To achieve active involvement, researchers are encouraged to consider how to support access to meetings and information and how to build relationships with public and patient members, so their involvement can be as effective as possible [1]. Patients and public members joining research teams generally follow an involvement process without the opportunity to influence the method of research co-production. A commonly used approach is to involve two individuals in groups undertaking research development, study management and study oversight [22]. Some research teams seek public and patient views on an individual study through a separate public or patient advisory group, instead or as well [18, 19, 23], but involving patient and public members across a programme of research is uncommon [24–26].

In contrast to the emphasis on co-production of research, little is written about methods to co-produce models for effective public and patient involvement. Rich descriptions of how involvement groups operate over time and how public members co-produce training to support their involvement are available [24, 27–30].However, these lack reproducible detail about how the groups were developed and the role of public members.

We aimed to identify a group of patients and carers to be involved in research and to support this group to define and establish structures and processes of working which they believed would best enable them to be involved in research. We believed this co-production approach would increase the number of public members involved in our research and improve the experience, for public and researcher collaborators. We also anticipated it would give research teams access to a wider range of patient and carer experiences to inform research development and implementation. This paper reports the method used by a group of patients and carers to develop and implement a model of involving public members in research over an 8 year period.

### Terminology

Within practice and discussion of involvement, one of the complexities is the language used to name the individuals and roles. When reporting our study, we use the term ‘service user’ to describe people who use health and care services as patients, former patients, prospective patients and people who care for others [1, 29, 31].

### Setting

We (HS with BAE and AP) were commissioned by the Welsh Government to evaluate implementation of the Chronic Conditions Policy in Wales [32] and wanted service users to be involved with this work. We did not have a preconceived idea about how to achieve this but wanted to establish a collaborative working relationship within the research team [1]. We obtained funding for 1.5 days per week of researcher time over 2 years to recruit service users, hold an initial workshop meeting and also to support the service users through the process of developing and implementing their involvement model. The funding also enabled us to reimburse expenses incurred by individuals and to offer an honorarium [1]. We offered an honorarium at the then-recommended rate of £65/half day or £130/day and reimbursed travel expenses, petrol costs and meals. We also covered overnight accommodation when this was required in order to undertake involvement activities. We then incorporated responsibility for facilitating the model into the researcher’s role and accessed the Involving People Network in Wales to support expenses and honoraria.

## Methods

We recruited individuals through two Welsh networks: one supporting public involvement in research (Involving People); the other coordinating support for people with experience of chronic conditions (the Long Terms Conditions Alliance-Cymru). The recruitment information is available at Additional file 1. We anticipated a response rate of between five and 25 people, based on previous experience of involving service users in research. We sought people diagnosed with, or caring for someone (adult or child) with, a chronic or long term condition, since carers and patients have shared and individual experiences of managing chronic illness. Relevant experience is considered an important aspect in enhancing effective involvement in research through collaboration [2, 3] (see Table 1). We provided information about the task, role and resources available, which the organisations disseminated through their networks and contacts. The information directed people to respond, if interested, to the respective organisation or to the lead author (BAE). She then telephoned each person to discuss the opportunity, confirm their interest and identify any requirements to facilitate their involvement, such as accessibility, diet, timings and training.

**Table 1:** Inclusion criteria for being involved in developing and implementing the model

Experience	Having a chronic condition and/or caring for someone with a chronic condition. Chronic conditions are those which are life-long, cannot usually be cured, limit quality of life and require ongoing management [37]
Knowledge	No knowledge of research was required but people needed an interest in being actively involved in research, as defined by INVOLVE [1]

We held one workshop for all interested service users to enable them to develop the involvement model from the start. This ran from 10.30 am-3 pm with lunch and refreshment breaks provided. The workshop format aimed to encourage discussion and consensus building using a modified Normative Group Technique [33]. It included group work sessions around open questions about factors facilitating and limiting involvement in research and how to design an accessible involvement model. Following feedback, we facilitated whole group discussion to discuss and confirm agreed items. The workshop was facilitated by BAE supported by a research colleague. They circulated among the groups, drawing out quieter members if appropriate, and becoming familiar with all attendees so discussions could be facilitated to provide optimum participation and reach consensus. The workshop programme is shown in Additional file 2.

We collected written records in order to describe the process of establishing the model and how it functioned between 2008 and 2015. We identified types of data to be collected in order to depict the ‘web of activities’ ([34] p2) that these data depicted and the iterative process which interwove the development and implementation processes. These were collected as the study proceeded and included: information to describe the workshop to devise the involvement model; notes and minutes of all meetings about development and implementation; a table of research activities members were involved with; notes of all other contacts with and support provided to service users and researchers.

We obtained informed, written consent from all service users and participating researchers to collect and use information. We reviewed documentary evidence chronologically to report the sequence and outcome of events, reading and re-reading the data and moving between different periods and source documents [34]. The purpose was to gain an overall impression and also to identify stages, key events and transitions in the story of developing and implementing the model [35].

Consent from a Research Ethics Committee was not required for this study. This was based on Health Research Authority guidance that it was not necessary because respondents were not identified through NHS sources and it was taking place in a non-NHS setting [36]. However, standards of ethical research were observed throughout.

## Results

### Characteristics of respondents

Twenty three service users expressed an interest in being involved: 19/23 service users were patients only; 1/23 was a parent of a child with a chronic condition; 3/23 were both a patient and a carer. The 22 patients reported having at least one chronic condition; 7/22 had two conditions; 3/22 had three conditions; 1/22 had five conditions; 1/22 said he lived with seven chronic conditions. One respondent, who was a patient, was also employed as a support worker. Reported conditions included arthritis, respiratory and heart conditions, diabetes and epilepsy, five of the six most common chronic conditions experienced in Wales. No one reported experience of stroke, the fifth most commonly treated chronic condition in Wales [37].

The 23 respondents came from 12 of the 22 Welsh counties (5/23 in northern Wales; 18/23 in southern Wales), living in urban and rural areas. Two thirds of respondents were women (15/23) and one third were men (8/23). This differed from the Welsh profile of patients with chronic conditions, which reports almost equal numbers of males and females with long term illnesses [37]. Respondents’ reported experiences of multiple conditions generally matched co-morbidity profiles and geographic distribution of chronic conditions patients in Wales. Respondents reported hearing about the opportunity through different channels, indicating that information was cascaded through a variety of networks to reach people with experience of chronic disease and interest in being involved in research.

Three respondents chose not to continue to participate after first telephone contact. One was suffering a relapse of her condition; another pulled out when her spouse was diagnosed with a terminal condition; and the third withdrew, saying she was no longer interested. The total number of service users who remained involved was 20.

Even though respondents experienced a range of different health conditions, their requirements for being involved in the workshop were similar. They asked for: meeting times which allowed for relaxed travel arrangements; good parking at venues; pre-confirmed meal times so they could manage medication; access to a quiet room or option to leave early if needed.

### How service users devised and developed the model

14/20 service users attended the workshop to discuss and develop an involvement model. Through group discussion, they identified principles which they agreed should underpin a model for collaborative involvement in research (Table 2). With these in mind, they agreed how they wanted to be involved in research identifying six components for the involvement model (see Table 3).

**Table 2:** Principles of effective involvement agreed by service users

Service users said that effective involvement should:	• Follow values and ways of working chosen by service users
	• Exhibit a culture which is inclusive and equally values the contributions of all participants including service users and researchers
	• Be well resourced (including travel and carer costs, support, training)
	• Be accessible (including venue, location, language, information, format)
	• Ensure mutual communication and feedback
	• Provide clarity about roles and responsibilities
	• Be a process which is relevant to all involved

**Table 3:** Components of the SUCCESS model

SUCCESS Panel made up of all SUCCESS members	
SUCCESS Steering Group meetings	
Opportunities for involvement in research activities open to all	
Representation and communication system between members	
Facilitator to coordinate involvement activities	
Supportive research environment	

Workshop notes were circulated to all service users (*n* = 20) and comments and amendments were invited, by email or telephone. No amendments were proposed. These principles and ways of working were reviewed at the next meeting, held 1 month later and agreed by all present to be the basis for the involvement model.

The model was named SUCCESS by members. SUCCESS was an acronym for Service Users with Chronic Conditions Encouraging Sensible Solutions. This was implemented at meetings and through email discussions in an iterative process as members’ experience and understanding of the research environment grew. It operated over 8 years until the Welsh research infrastructure was re-commissioned and thematically changed [38].

**Fig. 1:**
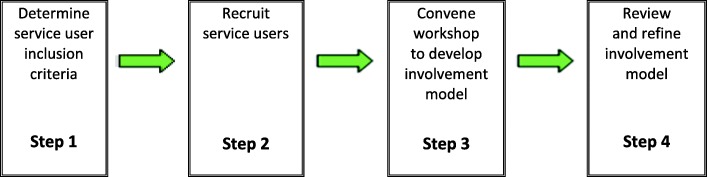
Process of developing the SUCCESS model

The process of developing the SUCCESS model is shown in Fig. 1.

### Components of the SUCCESS model

#### SUCCESS Panel

All service users were members of the Panel. Operation was through email, enabling communication between all SUCCESS members and with the group by the facilitator. All 20 inaugural members remained part of the SUCCESS Panel and received regular emails over the study period. The virtual forum enabled members to tailor their involvement as personal circumstances dictated. Some regularly contributed to discussions and involvement activities, others opted out periodically, while four were not active in any way because health and family circumstances prevented commitment.

#### SUCCESS Steering Group

Members asked to hold regular quarterly meetings, open to all in person or by Skype. They valued the personal contact of face-to-face meetings. These sessions, held 10 am-3 pm, were an opportunity for interactive discussion about research involvement and ways to fine-tune the SUCCESS model. For example, they selected a logo, agreed a mission statement and terms of reference, in order to implement the SUCCESS principles of effective involvement (Table 2). They also produced a leaflet and website. One new member was recruited after the information was circulated. Meetings and lunchtime conversations were also opportunities to share experiences and offer mutual support about research involvement and health care experiences which helped highlight any issues experienced by members and avoid or address challenges, such as skills and confidence in research meetings and communicating patient views to researchers, policy and clinical partners in projects. Attendance at SUCCESS Steering Group meetings fluctuated between seven and 14.

#### Involvement in research activities

The SUCCESS Panel was available to all researchers linked to the commissioned evaluation of the Chronic Conditions Policy in Wales [32]. All Panel members received information, circulated by the facilitator, about opportunities to be involved in research. Researchers, contacted by the facilitator and offered SUCCESS as a way of gaining public involvement, provided their information for distribution. Different routes of involvement were developed according to the type of research activity.

1. Researchers sought feedback about research proposals and ongoing studies by inviting comments on written information, distributed by email. They also attended Steering Group meetings to present and receive feedback on research proposals and results. For example, researchers who met SUCCESS members incorporated patient interviews to explore patient perspectives in a proposed study evaluating electronic records in ambulances, which gained NIHR funding [39]
2. The facilitator acted as a link between the research community and SUCCESS members by circulating opportunities to join research study teams and liaising between research teams and SUCCESS. These included the SAFER 2 study about referring older people, who fall and call 999, to a community falls service [40] and the PRISMATIC study about evaluating implementation of a risk prediction model in primary care [41].
3. SUCCESS members were involved in one-off research activities including attending and presenting at conferences, taking part in research development groups, piloting interview schedules and reviewing dissemination materials. The facilitator circulated information to the Panel who then took on activities, individually or collectively. For example: three SUCCESS members co-analysed patient stories about chronic conditions management services alongside researchers and practitioners [42]; SUCCESS members gave conference presentations on their work [43].

16/20 SUCCESS members were involved in at least one research activity over 8 years. The four non-active members, three of whom were patients and one a carer, said that health, family commitments or work arrangements were the reason. However, they all asked to continue receiving information and active members agreed the four should remain as members with the opportunity to contribute if they wished. The frequency of activities varied according to the work programmes of researchers, timings of funding calls and successful funding awards. Table 4 describes types of activities and numbers involved.

**Table 4:** Types and number of research activities undertaken by service users through the SUCCESS model 2008–2015

Type of research activity	Number of times activity occurred	Service user role	Number of individuals involved	Total number of times a SUCCESS member was involved^a^
Consultation to develop research ideas	5	Focus group participants considering options for research	9	41
Bid development^b^	12	Involvement in research development groups; commenting on research applications; named co-applicant	6	35
Research Management Group membership	6	Member of the group overseeing management and implementation of research projects	7	40
Intervention development	5	Group discussions with researchers to develop an intervention for a research study	13	31
Research tasks	4	Extracting data Piloting interview schedule Analysing data	9	33
Dissemination	16	Co-authoring abstracts and posters Making oral presentations Attending conferences	8	38

^a^Some individuals were involved more than once

^b^Bid development activity led to four proposals submitted for funding, three of which were successful

#### System for representation and communication

SUCCESS members established a system to enhance the breadth of patient and carer experience which individuals brought to research activities. Through a two-way process, individuals involved in research projects as SUCCESS members shared information about their activities with the Panel, who in turn provided contributions based on their experiences to enhance the input of each individual. They also reported to other health or community groups they belonged to. Email exchanges supplemented networking at Steering Group meetings. The aim was to enable SUCCESS members to reflect a patient voice that was wider than their personal experience, so that the SUCCESS model:

*is representing a wider experience of living with chronic conditions, not just a personal perspective. Experience of chronic conditions is the ‘common denominator’ (Minutes of SUCCESS Steering Group meeting, July 2010)*

#### Facilitator

The facilitator role was identified by service users as necessary to coordinate and support the operation of the model. The role included: distributing information to the SUCCESS Panel; convening SUCCESS Steering Group meetings; promoting the SUCCESS Panel to researchers; and recruiting service users for research activities. The facilitator also provided training within the SUCCESS meetings about research skills, structure and organisations within the research setting. All members had access to a range of courses provided through the Involving People Network, the network in Wales funded to support public involvement in research (http://www.wales.nhs.uk/sites3/home.cfm?orgid=1023). The facilitator provided briefing sessions about research studies for individuals who became involved in research opportunities as a SUCCESS member. A handbook was also prepared for each member (see Additional file 3). These aspects of the facilitator’s role enabled many of the principles of effective involvement (Table 2) to be achieved, such as: instilling inclusive and respectful values; resourcing involvement; ensuring accessibility and mutual communication. There were no instances where the principles were referred to in order to resolve tensions or poor practice. The facilitator had experience of involving service users in research, undertaking participatory group work and supporting and empowering individuals.

#### Research environment

The SUCCESS model operated in a supportive research environment. Senior academics were informed about best practice about involvement in research and briefed about the SUCCESS principles (Table 2). They encouraged service user involvement in research and demonstrated that they valued their contributions by: proactively seeking service users to join research projects; making involvement a standing agenda item in meetings to ensure service users contributed and to give status to their involvement; creating an accessible environment with non-jargon meetings, a welcoming atmosphere and meeting times to allow travel arrangements; directing research and administrative staff to involve and support service users. Barriers to involvement were identified by the facilitator and service users and addressed where possible, in discussion with senior staff where necessary, so that involvement processes were seamless. For example, Skype and phone participation was arranged for individuals unable to travel to meetings. Meeting times were altered to suit train travel times. Briefing sessions were held, with the facilitator or research staff, to provide extra information requested by service users. Administrative staff were encouraged to support and ease processes by booking carparking spaces and overnight accommodation, arranging suitable refreshments, processing paperwork and taking phone calls. The supportive, collaborative atmosphere of the research environment was noted and praised by service users.

## Discussion

We have described a method, co-produced by service users, to involve patients and carers in research. Our paper reports how the SUCCESS model was developed and how the components operated to enable public involvement in 218 research opportunities including developing research, research management meetings, research tasks and dissemination activities linked to different research projects.

Enabling service users to design the ways they are involved in research appears to have resulted in a distinctive and deliverable model underpinned by principles based on values and practical steps to support involvement. The method of involvement which they co-produced differs from other methods in several ways. Firstly it incorporated different levels and routes of involvement through the SUCCESS Panel and Steering Group meetings to enable involvement by written contribution, by group discussion, by joining research partnerships and by undertaking dissemination and engagement activities. These provided complementary ways to help members be involved in research. That most SUCCESS members were involved in at least one research activity suggests this range provided something accessible and of interest to almost everyone who joined SUCCESS. The model was also distinctive for creating ways for service users to gain mutual support, share information and build relationships. The emphasis on face-to-face contact and communication systems reflected the priority that these patients and carers gave to emotional needs and values, suggesting that these underpin the confidence, knowledge and skills that enable people to be actively involved in research. Despite calls for researchers to give feedback and reward to patients and public members to support their involvement, this can be overlooked or difficult to achieve [8, 44–46]. Additionally, informal support is reported to be as valuable as formal training for service users [47]. Thirdly, the method incorporated a system for reporting and communicating involvement activity between SUCCESS service users who undertook various research roles and the wider SUCCESS membership. This two-way communication was a distinctive feature of this novel involvement model: it aimed to communicate diverse patient and carer experience via SUCCESS members into research activities; and it was designed for service users to receive feedback on research progress and issues raised, relating to public involvement in research. The system for reporting and communication shares features of the outreach model of patient and public involvement [44] as a way of providing a conduit to and from a wider population. However, it benefited from the personal relationships formed between SUCCESS members. Having an opportunity to co-produce their involvement model enabled these individuals to jointly devise a system which combined their shared knowledge and enhanced their networks beyond the SUCCESS pool. This avoided the risks of tokenism cited by Wilson [44] who also observed that involved service users’ links with their population or patient group could weaken as their involvement experience grew. However, a common purpose and good relationships strengthen interaction among patients and public members [48]. These benefits of well developed relationships between service users and also with researchers, which are nurtured by a sustained period of involvement, may have outweighed the potential for involvement fatigue or the SUCCESS Panel’s perspective becoming more singular and less challenging for researchers. While some members could be more active than others, the size of the Panel increased the opportunity for a wider range of views to come forward. Our experience adds to the ongoing debate about whether people become too professionalised and lose their outside perspective over time [49].

We facilitated this method to co-produce an involvement model because we hoped it would increase the quality and quantity of involvement in our research. The tally of 218 separate and diverse research activities, involving people with various conditions as patients and carers, suggests that co-production added value to the model by incorporating features that enabled people to be involved in developing, implementing and disseminating research. We don’t report the experiences of research partners here. But it is likely that the model gave researchers an easy route to recruit people to their studies. Identifying service users is a reported challenge for researchers, limiting their ability and opportunity to identify people with relevant experience and to involve them in research [5, 22]. It is possible that researchers had greater confidence in a process incorporating the SUCCESS ‘brand’ because it suggested these service users had confidence, research awareness and experience to enable them to effectively contribute to research activities. Researchers report that skilled service users, who understand the timescales and demands of a research environment, help research partnerships to function more smoothly by supporting consensus in decisions about research development and implementation [5, 49, 50]. It is also possible that researchers perceived that the SUCCESS ‘brand’ indicated that these individuals represented more than an individual experience. Service users are often included in research because they are perceived to be able to talk on behalf of other people in a similar situation or can present a typical experience [49–53]. Their credibility comes from being ‘experience based experts’ [2]. However, people who seek opportunities to be involved in research may not be representative of the average patient or carer, even when their diagnosis or care experience is commonly shared [5, 54]. The SUCCESS members were a self-selected group and could not be typical of all patients and carers with chronic conditions. Nevertheless, their perspectives were based on their experience of living with illness and added to the views which informed the research activities they became involved with.

### Strengths and limitations

Data about development and implementation of the model, structure and operating processes provide a comprehensive description at a detail which allows it to be reproduced. This contrasts with the lack of detail and anecdotal quality of much research literature on this topic [3, 55, 56]. Experiences of those involved in research through the SUCCESS model are important in order to assess how well the model functioned. We do not report the views of service users and researchers who were involved nor assess its possible impact on research of involving service users through the model. However, we did interview all participants and these data are reported separately [57]. We have not assessed whether the model is sustainable in the long term.

Although the process of developing and implementing the SUCCESS model nurtured commitment and prolonged involvement from its service user members, it is unclear how this affects reproducibility in other contexts, with tighter budgets and in countries where existing patient/carer networks are not well developed. The iterative process of developing and implementing the model closely related to the individuals involved and the associated research programme. Membership did not change over the study period, except for one new recruit after leaflets were produced, which may have hindered development and dynamic exchange in the Panel. Involving people with fluctuating and deteriorating health means models must enable changing membership. This method of co-producing an involvement model enabled members to define what was relevant to their needs and would help them feel more informed, confident and skilled when contributing in research teams [58]. This is in line with other research reporting that self-organisation enables people to gain information, confidence and skills, develop ideas and potentially to exert more influence [45, 59].

Only one service user was a carer and three were both a patient and a carer. Researchers inviting SUCCESS involvement were seeking patient and carer perspectives and were informed of the experience mix among members. Although carers’ experiences differ from patients, we did not attempt to balance contributions from different subgroups or seek additional carer members. It is therefore possible that carers’ perspectives were not fully presented when researchers accessed views from SUCCESS members.

## Conclusion

Public and patient involvement is a routine element of health services research in order to improve accountability, rigour and relevance. Despite the emphasis on co-production in developing and undertaking research, methods through which public and patient members are involved are normally set by researchers. We supported patients and carers to co-produce the SUCCESS model of involvement in research. The model’s components, addressing their needs and priorities, led to sustained involvement in research over 8 years. Further work is needed to apply the model in different settings and assess impact of this method of involving people in research.

## Additional files

Additional file 1: Appendix 1: Title of data - Information sheet to recruit service users. Description of data – information which was circulated to recruit members to the SUCCESS model. (DOC 95 kb)

Additional file 2: Appendix 2: Title of data – Appendix 2: programme for the workshop to develop a model for involvement. Description of data – Table setting out the programme followed to deliver the workshop held to develop the SUCCESS model. (DOC 35 kb)

Additional file 3: Appendix 3: Title of data - Appendix 3 – Service user handbook developed and agreed by members of the SUCCESS model. Description of data – handbook provided to SUCCESS members. (PDF 546 kb)

## Abbreviations

NIHR: National Institute for Health Research
SUCCESS: Service Users with Chronic Conditions Encouraging Sensible Solutions
