# Supplementary material for: A co-produced method to involve service users in research: the SUCCESS model
Source: BMC Med Res Methodol. 2019 Feb 15;19:34. doi: 10.1186/s12874-019-0671-6 (PMC6377726; doi:10.1186/s12874-019-0671-6)
Supplement: Supplementary file 1 — Appendix 1: Title of data - Information sheet to recruit service users. Description of data – information which was circulated to recruit members to the SUCCESS model. (DOC 95 kb) [file 12874_2019_671_MOESM1_ESM.doc]

**Additional file 1: Information sheet to recruit service users**

**Involving people in research**

**about chronic conditions**

- Do you want to know whether government policies to help people in Wales with chronic conditions will benefit patients?
- Are you interested in research about services for people who have chronic conditions?
- Would you like to join a research network for service users and carers of people with chronic conditions?

This information sheet explains how research about Chronic Conditions policy in Wales is being planned and how service users and carers can be involved.

**Chronic Conditions policy in Wales**

A new policy for managing chronic conditions was published by the Welsh Assembly Government in 2007. Local Health Boards, who have responsibility for delivering chronic conditions services, have action plans setting out how they will treat people with chronic conditions and reduce people’s risk of getting them.

**Research and evaluation**

The new services will need to be evaluated to see how well they reach people and whether people benefit. A plan for researching and evaluating chronic conditions services has been developed. This **Framework for Research and Evaluation of Effectiveness (FREE)** was commissioned by the Welsh Assembly Government and produced by AWARD (the All Wales Alliance for Research and Development in Health and Social Care). FREE proposes a mix of research and evaluation projects taking place locally and nationally, to study the impact and the effectiveness of how programmes are delivered and how treatment works.

**What is planned?**

Local Health Boards will evaluate the services they provide. The National Public Health Service and Local Health Boards will collect information routinely gathered in the NHS to look at national and local trends. A study of how patients experience their care is being carried out by Swansea University to give a picture of services now (a baseline) against which future change can be compared. These, and other future research, all fit within the overall FREE Framework.

**Who will be involved?**

Researchers want people with experience of chronic conditions services to also be involved in research. When service users, patients and carers get involved, research can focus more clearly on users’ needs, so that:

- Research is about topics that are important to people
- Outcomes are relevant and important to people
- Methods are appropriate to the needs and circumstances of people

**Are you interested?**

We want people who use chronic conditions services or who care for people with chronic conditions to be involved in planning and undertaking research into chronic conditions services in Wales. Please consider joining the Chronic Conditions Research Network.

Members of our **Chronic Conditions Research Network** will:

- Oversee local and national research in Wales as part of the team managing FREE, the Framework for Research and Evaluation of Effectiveness
- Help decide how users are involved in chronic conditions research in Wales
- Be involved in future research projects

**What will it involve?**

- You will be invited to meetings about the research
- Travel expenses will be paid, to meetings held in different parts of Wales
- Frequency of meetings will depend on how involved you wish to be, but is likely to be about four times a year
- Information and training will be provided to help you take part

If you are interested in finding out more, please complete the slip below. If you express an interest now, but change your mind at a later stage, your decision will not be questioned.

-----------------------------------------------------------------------------------------------------------------

**Chronic Conditions Research Network**

Name ……………………………………………………………………………………………………………...

Address……………………………………………………………………………………………………………

……………………………………………………………………………………………………………………..

Tel ………………………………………………… Email ………………………..………………………

Have you been diagnosed with a chronic condition? Yes………. No ………

If so, please say what condition ……………………………………………………………………………

Do you care for someone with a chronic condition? Yes………. No ………

If so, please say what condition …………………………………………………………………………….

Please return to: Bridie Angela Evans, AWARD, Swansea University, Swansea SA2 8PP

Fax 01792 513423 or email [b.a.evans@swansea.ac.uk](mailto:b.a.evans@swansea.ac.uk)
