# Supplementary material for: A co-produced method to involve service users in research: the SUCCESS model
Source: BMC Med Res Methodol. 2019 Feb 15;19:34. doi: 10.1186/s12874-019-0671-6 (PMC6377726; doi:10.1186/s12874-019-0671-6)
Supplement: Supplementary file 2 — Appendix 2: Title of data – Appendix 2: programme for the workshop to develop a model for involvement. Description of data – Table setting out the programme followed to deliver the workshop held to develop the SUCCESS model. (DOC 35 kb) [file 12874_2019_671_MOESM2_ESM.doc]

Additional file 2: programme for the workshop to develop a model for involvement

| *Type of activity* | *Task* |
| --- | --- |
| Welcome and introductions | |
| Presentations by workshop facilitators | Presentations on:   - the chronic conditions policy - role of service users in research and evaluation |
| Group work (groups of 3-5 people) to identify good and bad practice when involving people in research, from the perspective of patients and carers | Small group discussion with comments recorded on flipchart to answer the questions:  1) *if patients and carers are involved in research, the process* ***should be****…*  2) *if patients and carers are involved in research, the process* ***should not be****…* |
| Feedback to whole meeting | Flipchart notes reviewed and then displayed on the wall for the remainder of the morning and over the lunch break |
| Group work (groups of 6-9 people) to brainstorm an ideal structure for involvement | Group discussion with comments recorded on flipchart to answer the question:  *How do we want everyone to be involved in this research project?* |
| Feedback to whole meeting | Flipchart notes reviewed and then displayed on the wall for the remainder of the morning and over the lunch break |
| Lunch | |
| Facilitated discussion to design and agree a model for involvement | Whole group discussion:   - to identify and agree a model for involvement - to identify next steps |
| Thanks and meeting closure: tea and depart | |
