# Supplementary material for: A co-produced method to involve service users in research: the SUCCESS model
Source: BMC Med Res Methodol. 2019 Feb 15;19:34. doi: 10.1186/s12874-019-0671-6 (PMC6377726; doi:10.1186/s12874-019-0671-6)
Supplement: Supplementary file 3 — Appendix 3: Title of data - Appendix 3 – Service user handbook developed and agreed by members of the SUCCESS model. Description of data – handbook provided to SUCCESS members. (PDF 546 kb) [file 12874_2019_671_MOESM3_ESM.pdf]

Appendix 3 – Service user handbook developed and agreed by members of the SUCCESS model

University of Wales Bangor
Cardiff University
Swansea University

AWARD All Wales Alliance for Research and Development in Health and Social Care
Cynghrair Cymru Gyfan ar Gyfer Ymchwil a Datblygiad mewn Iechyd a Gofal Cymdeithasol

Involving people in research about chronic conditions policy in Wales

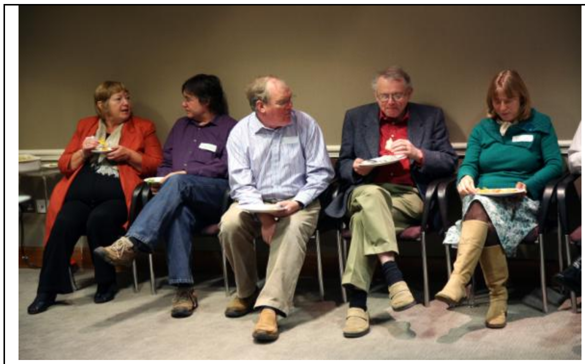

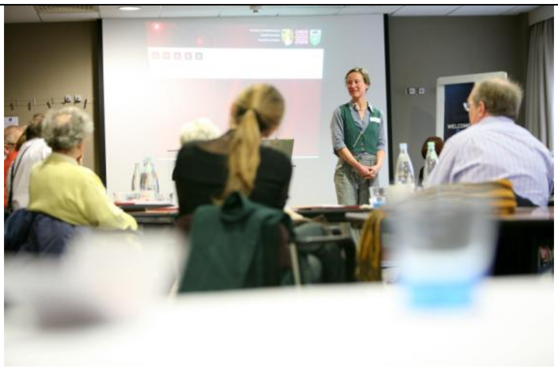

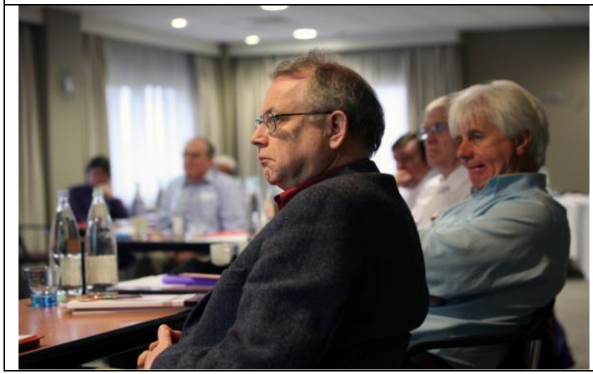

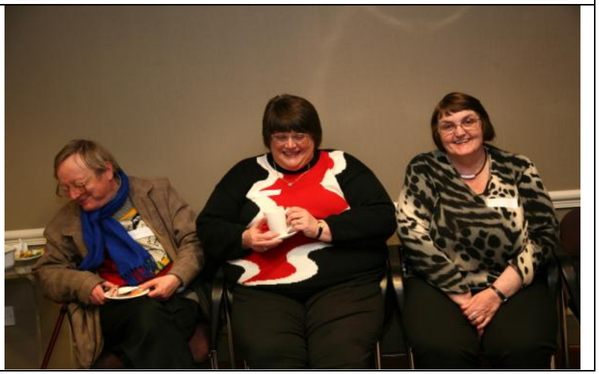

Handbook

CRC CYMRU
cydweithrediad ymchwil clinigol cymru
clinical research collaboration cymru

Contents

Contents 3
Introduction 4
Being involved in research 4
Research about the chronic conditions policy in Wales 5
Being involved in research linked to the chronic conditions policy in Wales 7
Project information sheet 8
Involvement model 10
Terms of Reference 11
Roles and responsibilities 13
Reimbursement of Expenses and Payment for Time 14
Reimbursement of expenses 14
Payment for time 15
Contact details 17
Glossary and sources of information 18

Involving People in Research
about the Chronic Conditions Policy in Wales

Introduction

This information pack is provided to service uses who are taking part in the project to involve people in research linked to the chronic conditions policy in Wales.

This pack has been prepared by the Project Researcher in consultation with service users. It contains useful information for people taking part in this project. The contents include items which service users have suggested are included in a pack such as this one because these are helpful when they get involved in research.

Being involved in research

Being involved in research means being part of a research team which plans, manages, carries out and presents research. This is a different role from being a research participant, where your views contribute data to help answer a research question.

Involving people in research is believed to lead to research which is more relevant to people's needs and concerns, is more reliable and is more likely to be used to improve health and social care services. Government policies and research funding organisations encourage researchers to include service users within their study teams.

Research findings can contribute to changing the way that services are delivered, for example by providing evidence about what works or doesn't work, or by evaluating a new service or treatment. But, being involved in research is a different task from being involved in planning and delivering services. This task is undertaken with organisations and individuals who commission and provide health and social care services.

Research about the chronic conditions policy in Wales

A new policy for delivering care to people with chronic and long term conditions was launched in Wales in 2007. It is an innovative strategy targeted at those who have - or who are at risk of developing -chronic conditions, in order to delay onset or deterioration; improve quality of life and ability to self-manage; and to reduce the burden on the NHS and social care services. Local Health Boards (LHBs) are responsible for implementing the initiative.

Alongside the new policy, AWARD (All Wales Alliance for Research and Development in Health and Social Care) was commissioned to develop an approach to conducting research and evaluation of the new chronic conditions management (CCM) policy. After consulting with policy makers, LHBs and other research or support organisations, AWARD presented a Framework for Research and Evaluation of Effectiveness (FfREE). This said that research about the CCM policy should answer the following questions:

1. **What is the overall impact of the CCM Programme?**
Trends in key performance will be monitored through health service management data which is already available. Information will be routinely collected at local (LHBs) and national (National Public Health Service NPHS) level throughout the study period 2007 – 2012 in order to assess outcomes from the CCM Programme. Types of performance indicators include: smoking numbers; prevalence of certain chronic diseases; emergency admission rates; number of Expert Patient Programme courses run.

2. **What is the impact of new services?**
Depending on the existing research evidence, research and evaluation will be undertaken to find out: a) whether a treatment or service is worth delivering; or b) how to deliver it, if it is already known to be an effective treatment. The first step therefore is to decide whether there is strong or weak research evidence for an innovation or service – that is, whether option a) or b) is followed.

3. **What is already known about the effectiveness of CCM services?**
This will involve an ongoing systematic review of studies of CCM interventions (ie. treatments and healthcare services), building on and updating the review undertaken by NPHS in 2005. The ongoing review by NPHS will focus on reviews of all interventions across the full range of chronic conditions, published since 1991 and will report annually. NPHS should report the results of this review annually.

4. **What CCM initiatives are planned in Wales?**
This will involve listing and describing CCM interventions and programmes which are in place or are planned, across Wales. These initiatives will be assessed against the objectives of WAG policy for CCM *"Designed to Improve Health and the Management of Chronic Conditions in Wales".* This work will build on the Welsh Audit Office exercise using a mix of data sources, including LHB documents, questionnaires and qualitative interviews.

5. **How do plans match policy and evidence?**
   This will involve a comparison of CCM interventions/programmes identified in no.4 above against the evidence about effectiveness identified through the review (no.3 above).

In 2007/09, AWARD was commissioned to implement the FfREE and to undertake research tasks 4 (in part) and 5 above. Meanwhile, the AWARD team received funding from CRC Cymru (Clinical Research Collaboration Cymru) to involve service users in developing and implementing FfREE.

Being involved in research linked to the chronic conditions policy in Wales

In 2007 AWARD received Special Project funding from CRC Cymru to enable service users and carers to be involved in the evaluation of CCM programmes in Wales. This was in line with a commitment in FfREE (the Framework for Research and Evaluation of Effectiveness) to involving patients, service users and carers in implementing and evaluating the CCM Programme in Wales. A Project Researcher was appointed to this two year, half time role, to develop, support and evaluate user involvement in CCM research and evaluation.

Project Aim

To effectively involve patients, service users and carers throughout the process of developing and implementing the Framework for Research and Evaluation of Effectiveness (FfREE) related to implementation of the Chronic Conditions Management Programme in Wales

Objectives

1. to identify appropriate groups, representatives and mechanisms for involving people in the development and implementation of the FfREE related to the CCM programme in Wales
2. to facilitate and support involvement in the development and implementation of the FfREE, through these mechanisms and with the guidance and support of Cynnwys Pobl:Involving People
3. to evaluate the process of involving people through the mechanisms developed during the study

Project information sheet

This information sheet was used when recruiting people to the project.

Involving people in research about chronic conditions

- Do you want to know whether government policies to help people in Wales with chronic conditions will benefit patients?
- Are you interested in research about services for people who have chronic conditions?
- Would you like to join a research network for service users and carers of people with chronic conditions?

This information sheet explains how research about Chronic Conditions policy in Wales is being planned and how service users and carers can be involved.

Chronic Conditions policy in Wales

A new policy for managing chronic conditions was published by the Welsh Assembly Government in 2007. Local Health Boards, who have responsibility for delivering chronic conditions services, have action plans setting out how they will treat people with chronic conditions and reduce people's risk of getting them.

Research and evaluation

The new services will need to be evaluated to see how well they reach people and whether people benefit. A plan for researching and evaluating chronic conditions services has been developed. This **Framework for Research and Evaluation of Effectiveness (FREE)** was commissioned by the Welsh Assembly Government and produced by AWARD (the All Wales Alliance for Research and Development in Health and Social Care). FREE proposes a mix of research and evaluation projects taking place locally and nationally, to study the impact and the effectiveness of how programmes are delivered and how treatment works.

What is planned?

Local Health Boards will evaluate the services they provide. The National Public Health Service and Local Health Boards will collect information routinely gathered in the NHS to look at national and local trends. A study of how patients experience their care is being carried out by Swansea University to give a picture of services now (a baseline) against which future change can be compared. These, and other future research, all fit within the overall FREE Framework.

Who will be involved?
Researchers want people with experience of chronic conditions services to also be involved in research. When service users, patients and carers get involved, research can focus more clearly on users' needs, so that:

- Research is about topics that are important to people
- Outcomes are relevant and important to people
- Methods are appropriate to the needs and circumstances of people

Are you interested?
We want people who use chronic conditions services or who care for people with chronic conditions to be involved in planning and undertaking research into chronic conditions services in Wales. Please consider joining the Chronic Conditions Research Network.

Members of our **Chronic Conditions Research Network** will:

- Oversee local and national research in Wales as part of the team managing FREE, the Framework for Research and Evaluation of Effectiveness
- Help decide how users are involved in chronic conditions research in Wales
- Be involved in future research projects

What will it involve?

- You will be invited to meetings about the research
- Travel expenses will be paid, to meetings held in different parts of Wales
- Frequency of meetings will depend on how involved you wish to be, but is likely to be about four times a year
- Information and training will be provided to help you take part

If you are interested in finding out more, please complete the slip below. If you express an interest now, but change your mind at a later stage, your decision will not be questioned.

Chronic Conditions Research Network

Name ………………………………………………………………………………………………….
Address………………………………………………………………………………………
…………………………………………………………………………………………………

Tel …………………………………. Email ………………………………………………

Have you been diagnosed with a chronic condition? Yes………. No ………
If so, please say what condition …………………………………………………………
Do you care for someone with a chronic condition? Yes………. No ………
If so, please say what condition …………………………………………………………

Please return to: Angela Evans, AWARD, Swansea University, Swansea SA2 8PP Fax 01792 513423 or email b.a.evans@swansea.ac.uk

Involvement model

This model for involvement was developed by members at their first meeting in September 2008 and agreed following circulation to the wider group. These notes formed the structure for the Terms of Reference which were subsequently agreed.

- Group is named Steering Group with purpose to steer information gathered from patients and carers
- Membership includes all patients and carers
- Group is supported by the researcher
- Group activity is task-led to provide focus and achievable outcomes; tasks* are linked to research needs, study protocol and workplan
- Agenda is set by the Study Team with group input
- Central meetings with flexible arrangements – varied locations, venues and times to suit all
- Option of subgroup or task-and-finish groups to encourage specific regional/topic/role involvement
- Alternatives means of communication other than meetings to allow everyone to participate – video, email, telephone, web discussion system
- Central register of members with preferred method of communication / participation listed
- Representatives to attend study team – 2 of 4 to attend, patient and carer if possible
- Patients/carers Steering Group to meet Study Team to: identify what each expects of the other; updates on progress

*At the third meeting of the Steering Group (February 2009), tasks were defined as:

- Meetings linked to research planning and strategy
- Participation in research projects

It was agreed that group members could also initiate tasks and research ideas.

Terms of Reference

Discussed at the second meeting of the Steering Group (October 2008) and then agreed

The project to involve people in research about chronic conditions policy in Wales is a two year project (1st January 2008 – 31st December 2009). It has three objectives:

- To identify a model for involving people in research about chronic conditions policy
- To support patient and carer's involvement in research about chronic conditions policy
- To evaluate the process and impact of involving people in research about chronic conditions policy

The final report will describe the process of involving people in research through this project and an evaluation of the impact on participants and research.

This information has been prepared and agreed by all Service User Steering Group members. It has been written so that all participants have an agreed list of roles and responsibilities to guide their involvement. It also means that others (eg. Research Team members and project commissioners) know what this group does and how it operates.

Being involved in research should be a joined-up process where the involvement of patients and carers is recognised and valued. Participants need to feel their contributions and involvement are valued and that the research is beneficial.

Role of Service User Steering Group

To steer information that is gathered from patients and carers and based on their experience of living with chronic conditions.

Membership should be open to people from all parts of Wales. Support should be provided to enable Group Members to undertake their role. This person will also coordinate meetings, provide and circulate minutes.

The Group will work in a task-led manner to provide focus and achievable outcomes. It will work to an agenda determined by research needs, study protocols and workplans set by the Research Team with Steering Group input.

Input to the group will be through a range of methods and in different formats to enable wide participation and ensure accessibility. These will include written and verbal communication in and outside meetings.

Representatives will attend the Research Study Team meetings and other meetings connected to the research and feedback to the Steering Group.

Funding for this project will run until December 2009 and the Steering Group will operate at least until that date.

The Steering Group will provide a means of involving patients and carers in research about delivering chronic conditions services. It is not a forum for influencing policy and service delivery directly and there are other organisations and groups which provide an opportunity to do this. It is hoped that research findings will inform future policy and services.

How the Steering Group operates
Communication and operation will be flexible to enable everyone to contribute regardless of geography, commitments and responsibilities, health and wellbeing, mobility and use of computers.

Every effort will be made to provide meetings in an accessible and comfortable environment.

People will be reimbursed for expenses incurred through participating. Guidelines for reimbursement are included in the project handbook. People will receive information in advance of meetings. The project researcher will provide some practical help to assist participation (for example, help to coordinate transport, booking accommodation, additional information).

The Group will operate in a way which ensures the confidentiality of all members; all information gained about each other through the Group will be treated with mutual respect, trust and confidentiality.

Meetings should be structured so that business is conducted efficiently within people's available time. At the same time, the tone should also be informal to encourage participation. All participants should feel their involvement and contributions are welcomed and equally valued. Participants should be encouraged to review the process of being involved and suggested amendments discussed.

Information should be presented in clear English, avoiding jargon and acronyms as far as possible.

Accounts of meetings and project activity should be circulated among all members of the Steering Group and/or Research Team through written or verbal reports. Those members attending meetings on behalf of others are responsible for ensuring updates are provided to all those they represent.

The Steering Group will operate a system of rotating chairman.

Roles and responsibilities

The Steering Group brings together all the patients and carers involved in this project. From its membership, individuals participate in identified research tasks. The Steering Group is also the forum through which service users' views are fed into research tasks.
The following process for participating in research tasks was agreed by group members at the third meeting in February 2009.

Opportunities to take part in meetings linked to research were shared among the group for the first few months of the project. This gave all the chance to take part but also meant there was no continuity between meetings. It was agreed that continuity was important and that people should be identified as permanent representatives of the group, to regular meetings. This would allow representatives to build up experience and knowledge so they can represent the views of patients and carers more effectively. They should also give comprehensive feedback to the wider group through verbal reports to supplement meeting minutes. Meeting representatives and responsibilities were set as follows:

- Two people and a reserve were identified for each meeting:

Research Study Team *(to discuss AWARD research)*
Jeremy Mead and Olwyn Cronin
Glynis Buckham (reserve)
WAG Research and Evaluation Meeting *(to discuss all research linked to the CCM policy)*
Mostyn Toghill and Jan Davies
Ron Woodall (reserve)
Demonstrators Sites Meeting *(to discuss research for the three LHBs chosen as sites to trial good practice)*
Judith Oades and Jeff Williams
Robert Harris-Mayes (reserve)

- Roles and responsibilities

To act as a delegate, attending on behalf of the group and communicating the views of these service users to the meeting you are presented at

To report back to the group, giving a more comprehensive account than usually provided in minutes. This could include highlighting decisions or discussions you consider to be important to the group, identifying areas where you need guidance for future meetings, reporting back any perceived impact of service user presence.

Minutes of the meetings will be circulated with the agenda for meetings of this group. Delegates' written comments can be added at this stage, for information to those unable to attend group meetings.

Reimbursement of Expenses and Payment for Time

The project *To Involve Patients and Carers in Research about Chronic Conditions Policy in Wales* is committed to ensuring that people receive reimbursement of expenses and payment for time in order that they feel able to participate when and as they want. The project has identified a separate budget, within the funding awarded to Swansea University by CRC Cymru, for reimbursement of expenses and payment for time.

Reimbursement of expenses and payment for time is administered by Swansea University and must comply with the university's financial regulations.

Reimbursement of expenses

Reimbursement is used here to mean being paid back what you have already spent (your expenses) because of your participation in a meeting or research activity connected to this project. You will be a member of the Steering Group and your participation will have been agreed by the Steering Group and the Project Researcher Angela Evans.

What expenses will be reimbursed?
The following expenses will be reimbursed. With all expenses claims, **you must provide a receipt.**

- **Travel costs** from your home to a meeting are paid at the Swansea University rate as follows:

40p per mile for the first 120 miles
13p per mile for additional distance travelled on any one return journey.

- We will cover **public transport costs and taxi travel** (with prior agreement) to and from the train or bus station.

- **Necessary overnight accommodation** will be paid by the project, with prior agreement. Swansea University will book accommodation in advance on request.

- **Meals and snacks** are generally provided at a meeting organised by the project. However, where you pay for meals, snacks and non alcoholic drinks whilst attending project business, the costs will be reimbursed within the following guidelines:

Project participation of 4-8 hours	£6
Project participation of 8-12 hours	£12
Project participation of more than 12 hours	£19

- **Car parking** costs are also reimbursed.

- **Alternative carer costs**: If you are a carer and must pay for someone else to cover your responsibilities in order to participate in the project, we will reimburse those costs.

- **Accompanying carer:** If you require a carer to accompany you, we will reimburse their travel and subsistence costs.

- **Personal assistant:** If you need a personal assistant to accompany you, we will reimburse their travel and subsistence costs.

When will expenses be reimbursed?
Reimbursement of expenses is managed by Swansea University. Your expenses will be reimbursed after the meeting or activity, provided a Swansea University expenses claim form has been completed and returned to Sherry Jenkins at Swansea University with all the relevant receipts or invoices. You will need to make the claim within two weeks of the event or activity they are relevant to. Once you have made a claim, you should be reimbursed within six weeks of it being received at Swansea University. We recognise this is not ideal but we must work within the university procedures. We can arrange for travel tickets and accommodation bookings to be made and paid for in advance if we have sufficient notice.

The university pays expenses into a claimant's bank account in the middle of a month. A paper copy detailing the payment is sent to you at the end of the month. We cannot guarantee that this payment will be in the month that you make your claim because of the several stages (and individuals) involved in processing the paperwork to the point where a payment is made.

How will expenses be reimbursed?
Your expenses will be reimbursed through Swansea University's finance department and paid directly into your bank account through BACS.

Either a Casual Payment Form (F-E.9.8) or an Expenses Claim Form (F-E.8.17) will require completion. If you are **only** claiming expenses, the Expenses Claim Form should be completed. If you are **also** claiming an honorarium, the Casual Payment Form should be completed **for both claims**.

Payment for time

Payment for time refers to an honorarium payment made when people contribute time for an identified project activity that has been agreed before hand.

Who can be paid for their time?
All members of the group who undertake a research task* will be offered an honorarium.

*At the third meeting of the Steering Group (February 2009), tasks were defined as:
- Meetings linked to research planning and strategy
- Participation in research projects

It was agreed that group members could also initiate tasks and research ideas.

Please note:
It is your responsibility, if you are receiving state benefits, to check with your local Job Centre Plus office about entitlement to receive additional payments before receiving an honorarium through this project. Due to regulations of the Department of Work and Pensions, if you are receiving income from certain State Benefits, you may only be entitled to accept limited payments or no payments at all in addition to your Benefits. More advice is also available from your local Citizen's Advice Bureau.

What payment for time will be made?
This project pays £130 a day or £65 per half day, in line with the figure recommended by Cynnwys Pobl:Involving People, the network which encourages user involvement in research in Wales.

How will payment be made?
Payment for participating in this project is managed by Swansea University and is made after the meeting or activity. A Casual Payment Form (F-E.9.8) will require completion. You can use this form to also claim expenses, if you are making both claims for the same activity. Payment is usually made directly into your bank account through BACS from Swansea University. Income Tax and National Insurance contributions will be deducted by Swansea University, if you earn more than a given amount each year. Additionally it is your responsibility to claim any tax entitlement.

If you have a comment or query about claiming expenses or an honorarium, please contact:
Sherry Jenkins
01792 602346
s.a.jenkins@swansea.ac.uk

All completed forms should be returned to:
Sherry Jenkins
School of Medicine
Swansea University
Freepost SWC4951
Swansea SA2 8ZZ

Contact details

Patient/carer contact details (if people are willing- Angela to check)

Angela Evans, Project Researcher, Swansea University
b.a.evans@swansea.ac.uk
Tel: 01792 295888

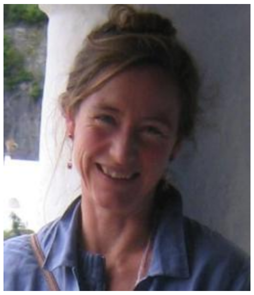

Sherry Jenkins, PA to Prof Helen Snooks, Swansea University
s.a.jenkins@swansea.ac.uk
Tel: 01792 602346

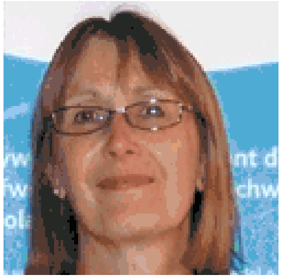

Helen Snooks, AWARD Regional Director and leader of research linked to the CCM policy in Wales
h.a.snooks@swansea.ac.uk
Tel: 01792 602346

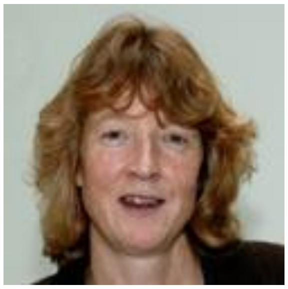

David Rea, Cynnwys Pobl:Involving People Associate Director
d.m.rea@swansea.ac.uk
Tel: 01792 295316

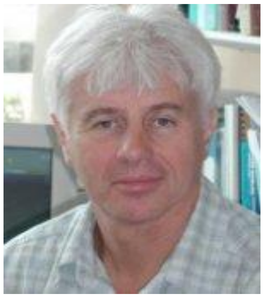

Glossary and sources of information

All Wales Alliance for Research and Development in Health and Social Care (AWARD)
AWARD was a multi-disciplinary team of researchers from Swansea, Cardiff and Bangor Universities which conducted and supported high quality research in health and social care. The network was funded by WORD (the Wales Office for Research and Development (part of the Welsh Assembly Government) for 2004-09. It was part of the Clinical Research Collaboration Cymru (CRC Cymru). AWARD was commissioned by the Welsh Assembly Government to support research linked to the chronic conditions policy in Wales. See www.awardresearch.org.uk for more information and copies of FfREE.

AWARD *Chronic Conditions Management Programme in Wales: Framework for Research and Evaluation 2007 – 12.* 2007, AWARD, Swansea. Available at http://www.awardresearch.org.uk/documents/CCM_final_FfRE_Dec_07.pdf

AWARD *Chronic Conditions Management Programme in Wales: Evaluation Handbook for Local Health Boards.* 2007, AWARD, Swansea. Available at http://www.awardresearch.org.uk/documents/LHB_CCM_Evaluation_Handbook.pdf

Chronic Condition
A condition which cannot be cured but may be controlled and which is often life-long and limiting in terms of quality of life. Other terms which are commonly used interchangeably are chronic disease, life-long disease, life-long condition, long-term disease, long-term condition.

Clinical Research Collaboration Cymru (CRC Cymru)
Established in 2006 by the Wales Office of Research and Development (WORD) to provide dedicated infrastructure to improve the quality, quantity, co-ordination, integration, inclusiveness and speed of health and social care research in Wales. See http://www.wales.nhs.uk/sites3/home.cfm?orgId=580

Cynnwys Pobl: Involving People
Network within CRC Cymru to support service user involvement within the strategy, development and implementation of health and social care research in Wales. For more information see www.involvingpeople.org.uk .

INVOLVE is a national advisory group, funded by the National Institute for Health Research (NIHR). Its role is to support and promote active public involvement in NHS, public health and social care research in order to improve the way that research is prioritised, commissioned, undertaken and disseminated. (Formerly Consumers in NHS Research). See http://www.involve.org.uk/.

Involvement

The process of enabling service users to take part in the research process. Levels of involvement are:

- *Consultation*: using service user views to inform decision making; for example, one-off meetings to collect views on a specific matter
- *Collaboration*: active ongoing partnership in the research process; for example, steering group membership, collaboration with researchers
- *Shared decision making*: another stage of partnership and one step on from collaboration, where all decision making is by mutual agreement and no one individual or representative group has a greater say than any other. Involvement is through the same processes as collaboration; for example, steering group membership
- *Service user control*: professionals are involved only at the invitation of service users; for example, a research study led and managed by service users

Local Health Board (LHB)

Local Health Boards (LHBs) in Wales are statutory bodies responsible for securing services to meet the health needs of the people in Wales. In October 2009, the 22 LHBs were reorganised into seven new Health Boards. The LHBs and Health Boards are listed at http://www.wales.nhs.uk/catorgs.cfm#22 .

Long Term Conditions Alliance Cymru (LTCA-C)

An alliance of organisations representing people with long term conditions which aims to encourage and facilitate service user empowerment and engagement. The Alliance has been working with the Welsh Assembly Government to develop and implement the CCM policy. See http://www.solnetwork.org.uk/newsdetails.asp?newsID=662

National Public Health Service for Wales (NPHS)

The National Public Health Service for Wales (NPHS) delivers a full range of public health services, seeking to:

- Improve the health and wellbeing of the people of Wales and reduce inequalities in health
- Protect against existing, new and emerging diseases and health threats
- Contribute to improvement in health and social care service

See www.nphs.wales.nhs.uk for more information.

The first review of research studies of CCM interventions was published by NPHS in 2006:

National Public Health Service for Wales *International Overview of the Evidence on Effective Service Models in Chronic Disease Management*. 2006, Welsh Assembly Government: Cardiff

Participant

Patient, service user, carer, relative of the deceased, employee, or member of the public, who consents to take part in a study. 'Participant' covers the person and any samples or data relating to the person that have not been completely anonymised.

Welsh Assembly Government Chronic Conditions Policy

The CCM policy Designed to Improve Health and the Management of Chronic Conditions in Wales is made up of two documents:

Welsh Assembly Government, *Designed to Improve Health and the Management of Chronic Conditions in Wales: An Integrated Model and Framework for Action*. 2007, Welsh Assembly Government: Cardiff. Available at http://www.wales.nhs.uk/documents/Chronic_Conditions_English.pdf

Welsh Assembly Government, *Designed to Improve Health and the Management of Chronic Conditions in Wales: Service Improvement Plan 2008-2011*
Welsh Assembly Government: Cardiff. Available at http://www.wales.nhs.uk/documents/serviceplane.pdf

An assessment of the prevalence of chronic conditions in Wales is available in:

Welsh Assembly Government *A profile of long-term and chronic conditions in Wales June 2006* Welsh Assembly Government: Cardiff.
